# Effect of indacaterol/glycopyrronium on ventilation and perfusion in COPD: a randomized trial

**DOI:** 10.1186/s12931-022-01949-3

**Published:** 2022-02-10

**Authors:** Dave Singh, Jim M. Wild, Dinesh Saralaya, Rod Lawson, Helen Marshall, Jonathan Goldin, Matthew S. Brown, Konstantinos Kostikas, Kristin Belmore, Robert Fogel, Francesco Patalano, Anton Drollmann, Surendra Machineni, Ieuan Jones, Denise Yates, Hanns-Christian Tillmann

**Affiliations:** 1 Medicines Evaluation Unit, University of Manchester, Manchester University National Health Service Foundation Trust, Manchester, UK; 2 Imaging Sciences, Department of Infection, Immunity and Cardiovascular Disease, POLARIS, University of Sheffield, Sheffield, UK; 3 Respiratory Clinical Trials Unit, Bradford Teaching Hospitals National Health Service Foundation Trust, Bradford, UK; 4 National Institute for Health Research, Sheffield Clinical Research Facility, Sheffield, UK; 5 MedQIA, Los Angeles, CA USA; 6 Center for Computer Vision and Imaging Biomarkers, Department of Radiological Sciences, David Geffen School of Medicine at University of California Los Angeles, Los Angeles, CA USA; 7 University of Ioannina Medical School, Ioannina, Greece; 8 Novartis Institutes for Biomedical Research, Cambridge, MA USA; 9 Novartis Pharmaceuticals Corporation, East Hanover, NJ USA; 10 Novartis Pharma AG, Basel, Switzerland; 11 Novartis Healthcare Pvt, Ltd, Hyderabad, India; 12 Novartis Institutes for Biomedical Research, Fabrikstrasse 2, Novartis Campus, 4056 Basel, Switzerland

**Keywords:** Chronic obstructive pulmonary disease, Hyperpolarized ^3^He gas magnetic resonance imaging, Indacaterol/glycopyrronium, Ventilation volume and perfusion volume, Ventilation/perfusion ratio, V/Q index

## Abstract

**Rationale:**

The long-acting β_2_-agonist/long-acting muscarinic antagonist combination indacaterol/glycopyrronium (IND/GLY) elicits bronchodilation, improves symptoms, and reduces exacerbations in COPD. Magnetic resonance imaging (MRI) of the lung with hyperpolarized gas and gadolinium contrast enhancement enables assessment of whole lung functional responses to IND/GLY.

**Objectives:**

The primary objective was assessment of effect of IND/GLY on global ventilated lung volume (%VV) versus placebo in COPD. Lung function, regional ventilation and perfusion in response to IND/GLY were also measured.

**Methods:**

This double-blind, randomized, placebo-controlled, crossover study assessed %VV and pulmonary perfusion in patients with moderate-to-severe COPD after 8 days of once-daily IND/GLY treatment (110/50 µg) followed by 8 days of placebo, or vice versa, using inhaled hyperpolarized ^3^He gas and gadolinium contrast-enhanced MRI, respectively. Lung function measures including spirometry were performed for each treatment after 8 days.

**Measurements and main results:**

Of 31 patients randomized, 29 completed both treatment periods. IND/GLY increased global %VV versus placebo (61.73% vs. 56.73%, respectively, least squares means treatment difference: 5.00% [90% CI 1.40 to 8.60]; *P* = 0.025). IND/GLY improved whole lung index of ventilation volume to perfusion volume (V/Q) ratio versus placebo; 94% (90% CI 83 to 105) versus 86% (90% CI 75 to 97; *P* = 0.047), respectively. IND/GLY showed a trend to improve diffusing capacity for carbon monoxide (DL_CO_) (+ 0.66 mL/min/mmHg; *P* = 0.082). By Day 8, forced expiratory volume in 1 s (FEV_1_) was increased by 0.32 L versus placebo (90% CI 0.26 to 0.38; *P* < 0.0001), substantiating earlier findings and providing evidence of assay sensitivity for this trial.

**Conclusions:**

IND/GLY improved lung ventilation assessed by ^3^He MRI after 1 week of treatment. This observation may provide mechanistic support for the symptomatic clinical benefit shown with IND/GLY in COPD.

Clinical trial registered with www.clinicaltrials.gov (NCT02634983).

**Supplementary Information:**

The online version contains supplementary material available at 10.1186/s12931-022-01949-3.

## Introduction

In evaluating pharmacodynamic (PD) treatment effects, spirometry has limited sensitivity and cannot detect regional airway responses to elucidate therapeutic mechanisms [1]. Magnetic resonance imaging (MRI) can non-invasively measure both regional lung ventilation and perfusion, which cannot be obtained by traditional lung function tests [2]. This is particularly relevant in the evaluation of novel therapies for chronic obstructive pulmonary disease (COPD), a complex, progressive lung condition classically associated with gas exchange impairment [3].

Of note, the correlation between changes in spirometric measures of lung function (e.g. forced expiratory volume in 1 s [FEV_1_]) after bronchodilator treatment and symptoms is poor [4], suggesting that other pathophysiological components beyond FEV_1_ improvement are responsible for the clinical benefits of pharmacological interventions. Heterogeneous ventilation throughout the lungs may play a role in this. The fixed-dose combination of long-acting β_2_-agonist/long-acting muscarinic antagonist (LABA/LAMA) indacaterol/glycopyrronium (IND/GLY) elicits bronchodilation, improves symptoms, and prevents exacerbations in COPD [5, 6], although the effects of long-acting bronchodilators on global ventilation and regional ventilation heterogeneity has not been fully elucidated. A recent publication from the CLAIM study in COPD patients selected for hyperinflation described improved pulmonary microvascular blood flow and regional ventilation following 14-day treatment with indacaterol/glycopyrronium, as measured by dynamic gadolinium-enhanced ^1^H MRI and phase-resolved functional lung (PREFUL)-MRI, respectively [7].

As ventilated lung volume is globally reduced in COPD, inhomogeneous ventilation is also present in this patient population and has been previously described [8]. Reduced ventilation, which can be a main driver of gas exchange impairment in COPD, leads to hypoxemia, resulting in compromised exercise tolerance, poor quality of life, and increased risk of death. Ventilation defects in COPD represent structural (emphysema and remodeled airways) and functional (bronchoconstriction, inflammation, mucus) pulmonary changes [9]. Reduced and inhomogeneous pulmonary ventilation may also lead to impaired ventilation/perfusion (V/Q) balance in the lung; termed V/Q mismatch or V/Q inequality in COPD patients [10]. This imbalance is patho-physiologically relevant in both mild [11] and advanced disease [12], and has been described as worsening during acute exacerbations [13].

Hyperpolarized gas MRI is a highly sensitive technique in which inhaled hyperpolarized gas (either ^3^He or ^129^Xe) provides the signal to generate high-resolution volumetric images of ventilation distribution in the lung [14]. Regional and global ventilation can be measured by this method as the entire lung is sampled at a spatial resolution with volumetric coverage hitherto unrealized with a gas ventilation imaging technique. Gadolinium contrast-enhanced perfusion ^1^H MRI can in parallel be employed in the same MRI examination to assess pulmonary perfusion [15]; thus, a combination of these two MRI assessments can be used to estimate the ratio of ventilation to perfusion as an index of the overall pulmonary V/Q balance [16].

The aim of this study was to investigate the effects of IND/GLY on lung ventilation and perfusion measured globally and regionally with hyperpolarized ^3^He (inert to lung tissue uptake) gas MRI and gadolinium-enhanced MRI, respectively. The global percentage ventilated lung volume (%VV) was the primary imaging derived endpoint and was hypothesized to increase with once-daily IND/GLY treatment.

## Methods

### Participants

Patients with COPD aged ≥ 40 years with post-bronchodilator FEV_1_ ≥ 30–< 80% predicted and post-bronchodilator FEV_1_/forced vital capacity [FVC] ratio < 0.7, a smoking history of ≥ 10 pack-years), and without gross emphysema (< 25% emphysematous total lung changes in high-resolution computed tomography [HRCT] at screening) were included. Full inclusion and exclusion criteria are available in Additional file 1.

### Study design

This study (NCT02634983, EudraCT 2013-004461-13) was a randomized, double-blind, placebo-controlled, multicenter, two-period, crossover study, in which the effect of 7 days of IND/GLY therapy on lung ventilation and perfusion, and 8 days of IND/GLY on other lung function measurements, versus placebo were assessed.

**Fig. 1:**
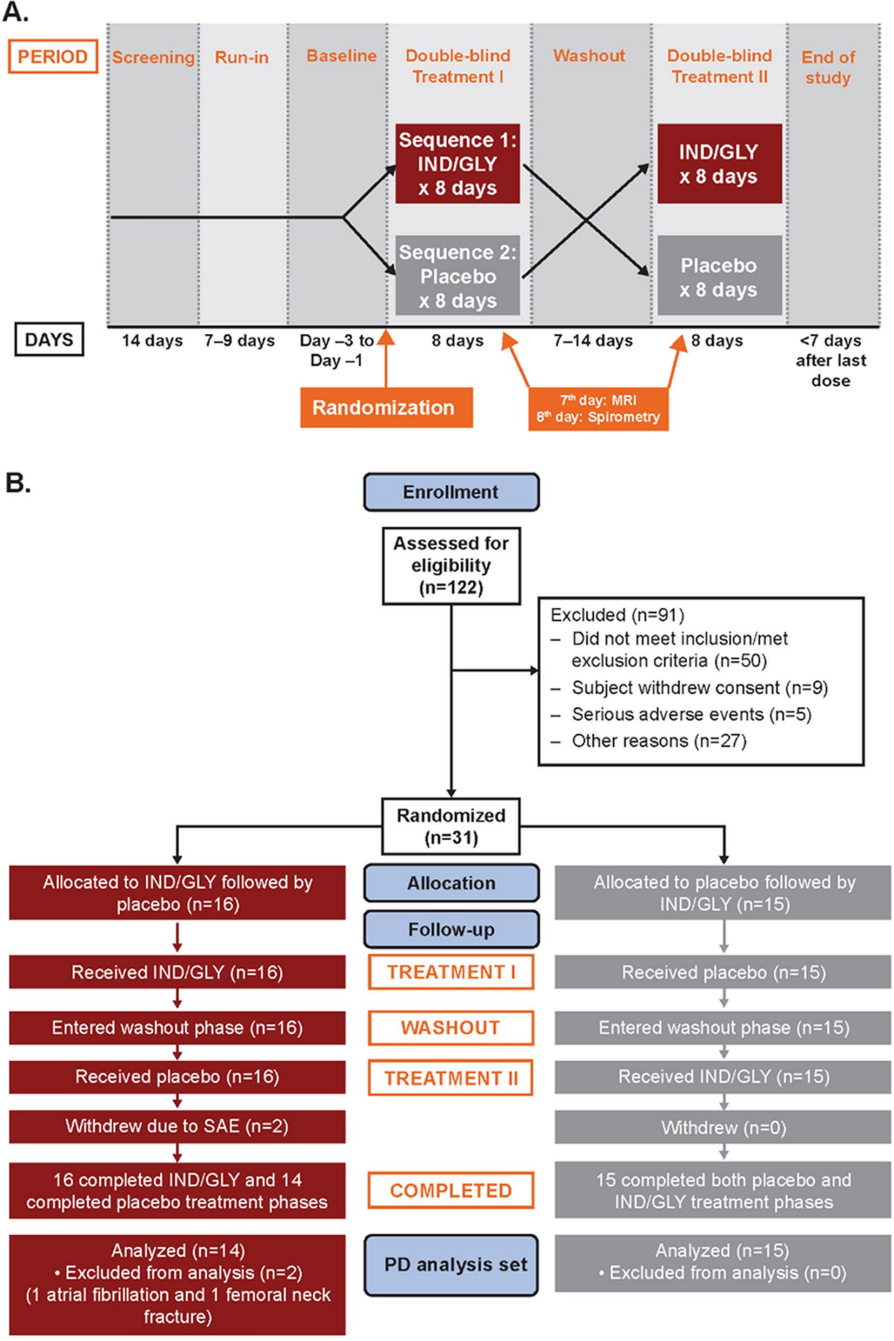
**A** Study design. This was a randomized, double-blind, placebo-controlled, two-period crossover study of approximately 8 weeks and consisted of 7 periods. **B** Study disposition. A total of 122 patients were screened, of whom 31 were randomized to one of two treatment sequences. Two patients did not complete both treatment period I and II due to SAEs (one atrial fibrillation and one femoral neck fracture). Screen failures relating to inclusion/exclusion criteria primarily included clinically significant electrocardiogram abnormalities, abnormal computed tomography scan, ineligible pulmonary function test results, or COPD exacerbation. *IND/GLY* indacaterol/glycopyrronium, *MRI* magnetic resonance imaging, *PD* pharmacodynamics, *SAE* serious adverse event

Participants were recruited from three hospitals in the UK (Bradford, Manchester, and Sheffield). High resolution computed tomography (HRCT) screening was conducted at each clinical center, while magnetic resonance imaging (MRI) was conducted at a single center at the University of Sheffield. Patients were randomized to receive either once-daily IND/GLY (110/50 μg) in treatment period I for 8 days, followed by placebo for 8 days in treatment period II, or the same treatments in the reverse sequence, at an allocation ratio of 1:1. Treatment periods I and II were separated by a 14-day washout period (Fig. 1A). Further details are available in Additional file 1.

Approval was obtained from the Institutional Review Boards/Independent Ethics Committees of participating sites.

### Study objectives

The primary objective of this study was to assess global %VV using hyperpolarized ^3^He gas MRI after IND/GLY treatment compared with placebo. Secondary objectives included regional %VV, pulmonary perfusion, lung function, and diffusing capacity for carbon monoxide (DL_CO_), as well as estimation of an MRI based index of the V/Q ratio derived from global ventilation and perfusion volume estimates in response to dual bronchodilation with IND/GLY as an exploratory endpoint.

### Study procedures

HRCT evaluated by a central reader was used to assess the extent of emphysema (< 25% total lung volume) at screening. Hyperpolarized ^3^He MRI was employed to assess global and regional %VV 2 h post-dose on Day 7 of each treatment period. Dynamic gadolinium-enhanced ^1^H MRI was performed during the same MRI session to assess global and regional pulmonary perfusion reported as the percentage of perfused lung volume. Spirometry and DL_CO_ measurements were collected 2 h after the last study dose (Day 8) of each treatment period. The assessments were split over 2 days to reduce the burden on patients during assessment days. More details, including MRI acquisition parameters, are provided in Additional file 1.

### Statistical analysis

A sample size of 28 completers (14 patients per treatment) was estimated to provide ≥ 80% power to detect a 5% improvement in global %VV using a 2-sided test at the 10% significance level, assuming a 7% standard deviation (SD). All primary and secondary analyses were performed in the PD population, i.e. all patients with evaluable PD parameter data and no major protocol deviations. %VV on Day 7 was analyzed using a mixed-effects model, which included crossover sequence, period, and treatment as fixed effects, and patient factor as a random effect. The study protocol pre-specified that *P* < 0.1 conferred statistical significance for this exploratory mechanistic evaluation. The statistical analyses were conducted by Novartis Institutes for Biomedical Research, Switzerland and Novartis Healthcare Pvt. Ltd., India using SAS software version 9.4. Further statistical information is provided in Additional file 1.

## Results

### Participant characteristics

This study was conducted between June 3, 2016 and September 26, 2017, and all patients provided written informed consent. Of 122 patients screened for inclusion, 31 patients were randomized to one of the two treatment sequences (Fig. 1B). The majority of screen failures related to inclusion/exclusion criteria (*n* = 50), including clinically significant electrocardiogram abnormalities, abnormal computed tomography scans, ineligible pulmonary function test results, or COPD exacerbations. Twenty-nine patients completed the study.

The median (range) age of patients was 68.0 (53–76) years. All patients were Caucasian with moderate-to-severe COPD, and 51.6% of participants were male (Table 1). The mean (SD) predicted post-bronchodilator FEV_1_ of the participants at screening was 53.1% (11.68), and the mean (SD) % lung emphysema measured by HRCT was 10.14% (7.35).

**Table 1:** Baseline participant demographics and characteristics (PD analysis set, *n* = 31)

Parameter	All patients (*n* = 31)
Age (median [range]), year	68.0 (53–76)
Sex, *n* (%)	
Male	16 (51.6)
Body mass index (mean [SD]), kg/m^2^	26.53 (4.49)
Race, *n* (%)	
Caucasian	31 (100.0)
Severity of COPD—airflow limitation^a^, *n* (%)	
Moderate (GOLD 2)	21 (67.7)
Severe (GOLD 3)	10 (32.3)
Lung function (post-bronchodilator^b^) (mean [SD])	
FEV_1_, % predicted	53.1 (11.68)
FEV_1_/FVC, %	46.4 (8.82)
Residual volume, L	2.9 (0.8)
Inspiratory capacity, L	2.2 (0.56)
Ventilated lung volume (mean [SD]), %	55.42 (19.85)
Degree of emphysema at screening (mean [SD]), %	10.14 (7.35)

*COPD* chronic obstructive pulmonary disease, *FEV*_*1*_ forced expiratory volume in 1 s, *FVC* forced vital capacity, *GOLD* Global Initiative for Chronic Obstructive Lung Disease, *PD* pharmacodynamics, *SD* standard deviation

^a^As per GOLD 2015 guidelines

^b^Lung function measurements were taken 60 min post-bronchodilation

### Efficacy outcomes

#### MRI assessments

**Fig. 2:**
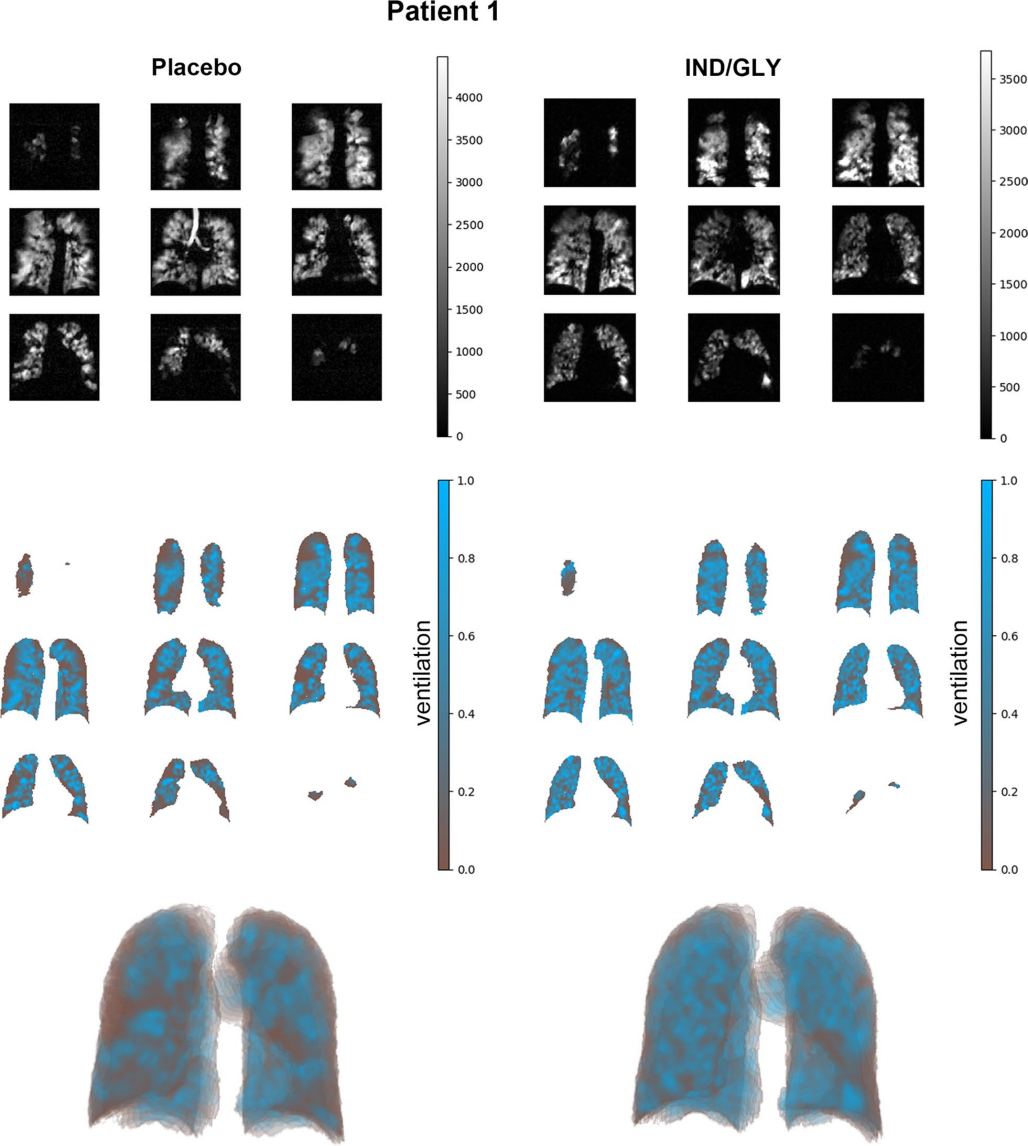
Distribution of ^3^He within the lung by MRI: quantitative percentage ventilated lung volume assessment for sample Patient 1 (additional images and video are available in Additional file 1: Fig. S1). Shown are example ^3^He MRI image segmentations of the lungs acquired during the placebo and treatment periods from a patient demonstrating the effect of IND/GLY compared with placebo. Upper panels: Source images showing the distribution of hyperpolarized ^3^He gas within the lung. 2D coronal slices from back to front (upper left to lower right) were acquired for volumetric assessment after 7 days of placebo and IND/GLY treatment in the respective treatment periods. Center panels: Image segmentations with ventilated lung shown in blue and unventilated lung shown in brown (2D slices). Lower panels: 3D volume rendering of the image segmentations. *IND/GLY* indacaterol/glycopyrronium, *MRI* magnetic resonance imaging

**Fig. 3:**
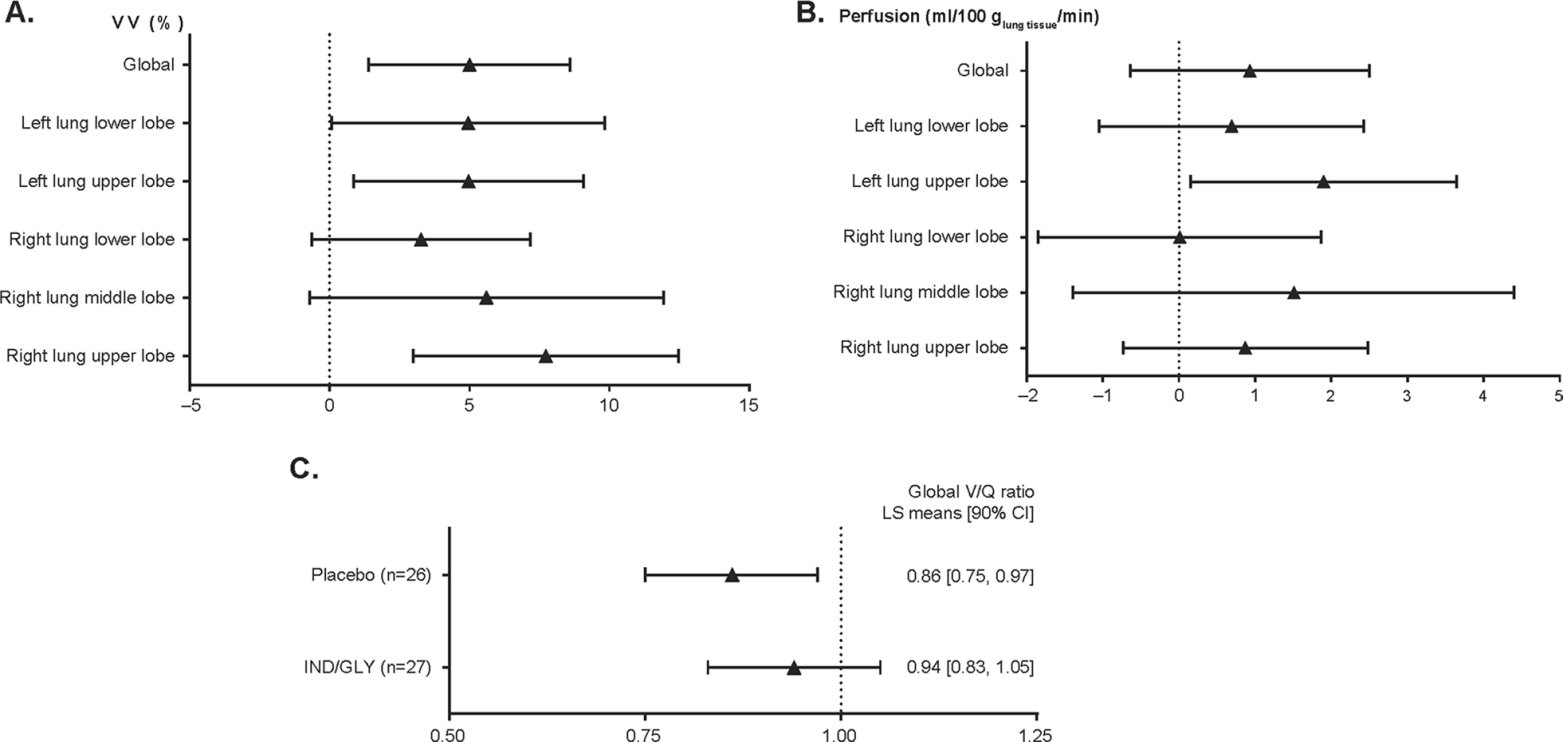
Effect of indacaterol/glycopyrronium treatment on measurements of lung ventilation and perfusion—PD set. Absolute difference in MRI-derived **A** % VV, **B** pulmonary perfusion (mL/100 g_lung tissue_/min) and **C** V/Q after 1 week of treatment compared with placebo in the PD analysis set. Parameters were calculated using a mixed-effects model including crossover sequence, period, and treatment as fixed factors, and patient as a random effect. Data are presented as LS mean treatment differences ± 90% CI compared with placebo. *CI* confidence interval, *LS* least squares, *PD* pharmacodynamics, *VV* ventilated lung volume, *V/Q* ventilation volume/perfusion volume

Color maps with 2D and 3D rendering of a sample patients’ ventilation MRI dataset depict treatment response to IND/GLY when compared with placebo and are shown in Fig. 2 and Additional file 1: Fig. S1 [17]. Global %VV was significantly increased in patients after 7 days of IND/GLY treatment versus placebo (least squares [LS] means [90% CI] 61.73% [56.16 to 67.30] vs. 56.73% [51.07 to 62.39], respectively; LS mean treatment difference 5.00% [90% CI 1.40 to 8.60; *P* = 0.025], Fig. 3A). Regional assessments showed similar ventilation improvements in all lobes following dual bronchodilation when compared with placebo (Fig. 3A), with some regional treatment effects reaching statistical significance (i.e. ventilation of the left [*P* = 0.035] and right [*P* = 0.029] lungs, and the upper lobes of the left [*P* = 0.049] and right [*P* = 0.010] lungs). 3D-rendered videos of the entire ventilated lung volume to illustrate improvement in global %VV with IND/GLY versus placebo are provided in Additional file 1.

In this patient population, global lung perfusion was unchanged by dual bronchodilation when compared with placebo (LS means [90% CI] 13.96 [12.50 to 15.41] vs. 13.03 [11.56–14.50] mL/100 g_lung tissue_/min, respectively; LS mean treatment difference 0.93 mL/100 g_lung tissue_/min [90% CI − 0.64 to 2.50; *P* = 0.323]. Similar non-significance was also observed for regional measurements of perfusion (Fig. 3B).

Global ventilation and perfusion volume fractions were used to estimate the MRI-based V/Q ratio of the whole lung as an exploratory endpoint. The V/Q ratio, expressed here as %, was significantly rebalanced following treatment with IND/GLY versus placebo. V/Q ratio was 94% (90% CI 83 to 105) following IND/GLY when compared with 86% (90% CI 75 to 97) after placebo (LS mean treatment difference 8% [90% CI 1 to 15; *P* = 0.047]) (Fig. 3C).

#### Spirometry assessments and lung volumes

**Fig. 4:**
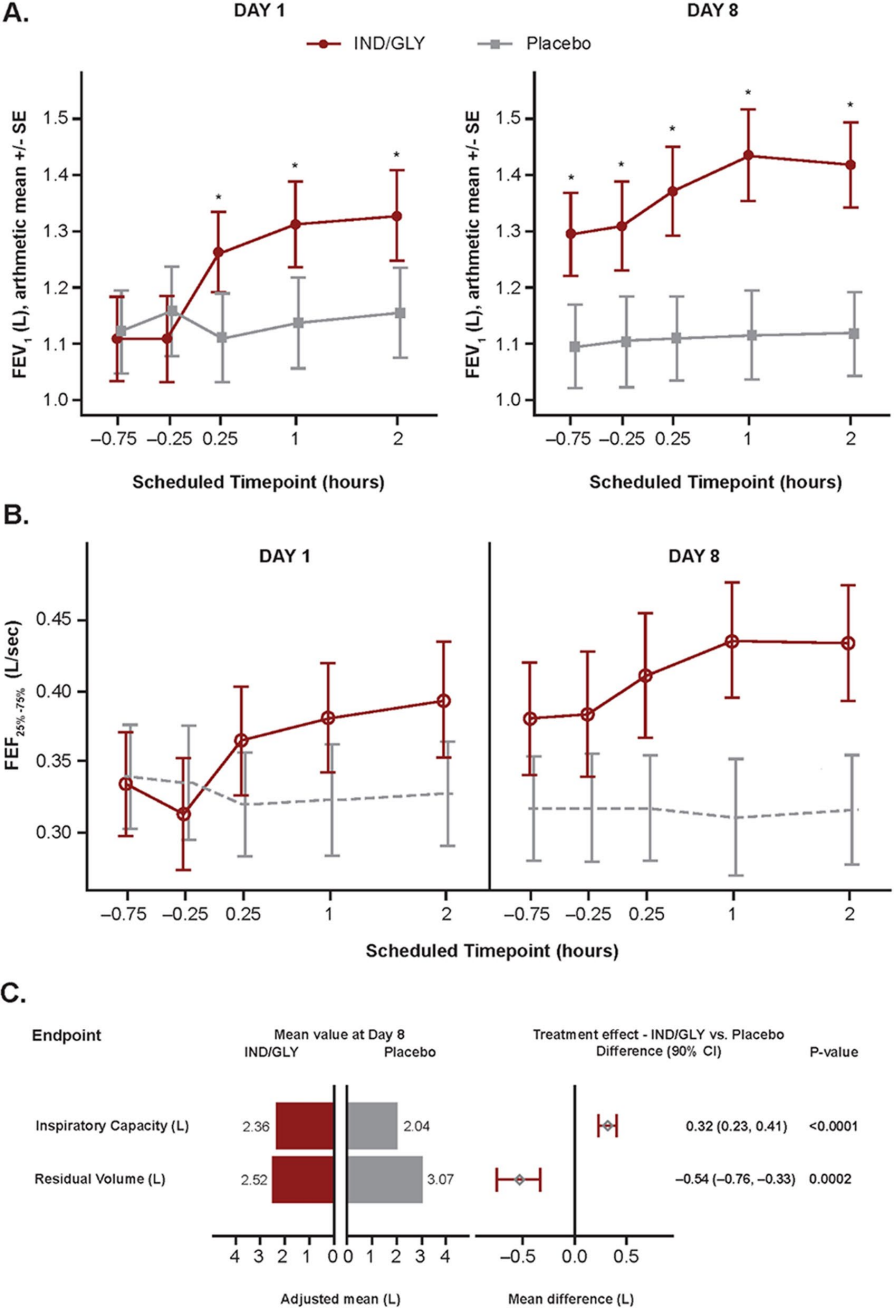
Effect of IND/GLY treatment on lung function—PD set. Analysis of IND/GLY treatment on **A** FEV_1_ and **B** FEF_25–75%_ over time. Measurements were obtained 0.75 and 0.25 h pre-dose, and 0.25, 1 and 2 h post-dose on Day 1, and 0.75 and 0.25 h pre-dose and 0.25, 1 and 2 h post-dose on Day 8. **C** The effects of 8 days of IND/GLY versus placebo treatment on residual volume and inspiratory capacity are shown. Data were analyzed using a mixed-effects model including crossover sequence, period, treatment, and time as fixed factors, and patients as a random effect. *P < 0.0001. *FEV*_*1*_ forced expiratory volume in 1 s, *FEF* forced expiratory flow, *IND/GLY* indacaterol/glycopyrronium, *SE* standard error

FEV_1_ and forced expiratory flow at 25% to 75% of forced vital capacity (FEF_25–75%_) were measured at intervals pre- and post-dose on Day 1 and 8 of each treatment period. IND/GLY induced lung function improvements over placebo as early as 15 min post-dose on Day 1. By the final spirometry assessment on Day 8, IND/GLY treatment had improved post-dose mean (SD) FEV_1_ from 1.11 L (0.41) at baseline to 1.42 L (0.42). When compared to the placebo, this represented a treatment difference of 0.320 L with IND/GLY on Day 8 (LS means: 1.11 L vs. 1.43 L, respectively; LS mean treatment difference 0.32 L [90% CI 0.26 to 0.38; *P* < 0.0001] at 2 h post-dose). The differences observed in FEV_1_ and FEF_25–75%_ between the two treatments were significantly different at all post-dose time-points on Day 1 and all time-points on Day 8 (*P* < 0.0001, Fig. 4A and B). Similarly, IND/GLY significantly increased inspiratory capacity and reduced residual volume (LS mean treatment difference 0.32 L [90% CI 0.23 to 0.41; *P* < 0.0001], and − 0.54 L [90% CI − 0.76 to − 0.33; *P* < 0.0001], respectively) when compared with placebo (Fig. 4C). As a measure of gas exchange, IND/GLY increased DL_CO_ versus placebo (LS mean treatment difference 0.66 mL/min/mmHg [90% CI 0.04 to 1.27; *P* = 0.082]) after 8 days of treatment.

Correlation analyses confirmed that by limiting inclusion based on HRCT assessment of the degree of emphysema to be less than 25% of the total lung, no impact on the treatment effects of IND/GLY for global percentage ventilated lung volume (r^2^ = 0.08), inspiratory capacity (r^2^ = 0.08), or FEV_1_ (r^2^ = 0.02) were observed (Table 2).

**Table 2:** Correlation between % emphysema at screening with measurements of ventilation and lung function (intra-individual difference between indacaterol/glycopyrronium and placebo)

Parameter 1	Parameter 2	r^2^ value
Percentage ventilated lung volume at Day 7	Extent of emphysema at screening (%)	0.08
Inspiratory capacity at Day 8 (L)	Extent of emphysema at screening (%)	0.08
Forced expiratory volume in 1 s on Day 8 (L)^a^	Extent of emphysema at screening (%)	0.02

^a^Measured 2 h post study medication

Further post-hoc analyses demonstrated that intra-individual differences in residual volume (r^2^ = 0.01) and inspiratory capacity (r^2^ = − 0.39), or FEV_1_ (r^2^ = 0.06), were not associated with change in %VV; however, the study was not powered to yield any conclusive data on the relationship of the imaging assessments with traditional lung function testing.

### Safety findings

The overall safety profile for IND/GLY was comparable with placebo. The number of patients affected by at least one adverse event (AE) was similar between patients receiving IND/GLY and placebo (16.1% vs. 12.9%, respectively) (Additional file 1: Table S1). IND/GLY was well tolerated, and there were no new safety findings for IND/GLY.

## Discussion

In this randomized controlled trial, once-daily inhaled IND/GLY significantly increased regional pulmonary ventilation and improved whole-lung-MRI-measured V/Q matching after 7 days of treatment in patients with moderate-to-severe COPD. IND/GLY increased %VV by 5%. Similar improvements in ventilation with IND/GLY treatment have also been reported using other MRI methodologies [7]. Importantly, ventilation defect percentage (VDP = 100%–%VV) is significantly associated with quality of life in patients with COPD, as measured by the St. George’s Respiratory Questionnaire. VDP is also independently correlated with quality of life and disease control in patients with asthma [18]. A recent communication describes that a change of 2% in %VV (expressed as ventilation defect) as determined by hyperpolarized gas MRI is the minimally clinical important difference (MCID) in asthma patients [19]. With all limitations considered in terms of comparability between asthma and COPD, it is hence possible that the observed increase in ventilated lung volume of 5% (absolute) in this study is beyond the MCID for this parameter.

In line with previous studies, FEV_1_ [5, 20], residual volume, and inspiratory capacity [21] were significantly improved following treatment with IND/GLY. However, these parameters did not correlate well with %VV in this study. While the study was not powered to establish such correlations, the results suggest that advanced and sensitive lung volume assessments like hyperpolarized gas MRI may capture different aspects of the PD of bronchodilators to spirometry and traditional lung volume assessments in patients with COPD. We speculate that this potential ‘limitation’ of spirometry is a possible mechanism to explain the weak correlation often observed between FEV_1_ and symptoms or health status [3]. Because these methodologies are not equivalently sampling the airways, overall correlation between the functional MRI assessment and spirometric measures may require a larger study to perform such meaningful comparisons.

The consequences of compromised ventilation and perfusion, and the relationship between these two parameters in COPD is well known [22]. In markedly hyperinflated COPD patients a reduction of such hyperinflation by dual bronchodilation with IND/GLY has also been shown to be associated with pulmonary perfusion improvement, particularly in the pulmonary microvasculature [7] potentially triggered by a reduced pressure of hyperinflated airways on the microvasculature.

In the COPD patient population of this study, not characterized by marked hyperinflation, the numerical increase in pulmonary perfusion was not statistically significant (Fig. 3B). However, arguably just as important as an increase of microvascular blood is the V/Q match, the importance of which has been reiterated in an editorial by Watz [23] commenting on the results of Vogel-Claussen et al. [7].

An unbalanced distribution between ventilation and perfusion is a leading driver of hypoxemia in COPD. Such imbalances can be amplified during COPD exacerbations and can worsen with disease severity [24]. V/Q mismatch is associated with various pathophysiological changes such as progressive airflow limitation, emphysematous destruction of the pulmonary capillary bed, heterogeneous alveolar hypoventilation, substantial perfusion of under-ventilated areas, and consequent physiological shunt [25]. V/Q mismatch has been estimated previously in patients with COPD receiving standard-of-care therapy using a similar MRI methodology to that described herein [26], but this study reports for the first time the effect of the dual bronchodilator IND/GLY on the derived index of lung V/Q as measured by MRI. Therefore, the observed improvement in global V/Q, which was likely driven by IND/GLY-mediated ventilation improvements, adds to our knowledge of this treatment response from a mechanistic perspective. The finding of a statistically significant increase in global %VV, and that global V/Q matching was improved following IND/GLY, can have important clinical implications for patients. Increased ventilation with IND/GLY may facilitate increased oxygenation reserves for improved exercise tolerance and reduced dyspnea [27]. However, this study was not designed or powered to determine the relationship between improvements in V/Q mismatch and clinical benefits for COPD patients. Nevertheless, it is known that IND/GLY improves COPD symptoms and lung function, and the ^3^He and ^1^H MRI-demonstrated changes in ventilation and perfusion may provide a mechanism to better explain the established clinical profile of dual bronchodilation. Consistent findings from a slightly smaller study using ^129^Xe MRI support this notion [28].

DL_CO_, as a functional measure of alveolar capillary gas transfer efficiency [29], is associated with physical function in COPD patients, with decreased DL_CO_ predicting clinically relevant declines in 6-min walk distance tests [30]. Although DL_CO_ can be affected by ventilation [31], this measurement may lack the sensitivity to measure intra-individual variations. In this study, an improvement in DL_CO_ with IND/GLY was observed. Although the observed improvement in DL_CO_ was statistically significant (*P* < 0.1) for informing on the trend in treatment response only, it did not achieve the accepted standard for statistical analysis of *P* < 0.05. The study was not powered to detect a treatment effect of IND/GLY on DL_CO_ versus placebo and therefore the authors caution against its over-interpretation.

IND/GLY was well tolerated in this patient population, with a comparable number of AEs affecting patients receiving IND/GLY and placebo.

This study carries some limitations. It was performed in a small patient population, which may limit the applicability of these findings to wider populations. A maximum threshold of 25% emphysema was applied for inclusion in this study, which precluded assessment of IND/GLY in patients with gross emphysema. Although the fast onset of bronchodilatory action of IND/GLY facilitated a short study duration, a study assessing the effects of long-term dual bronchodilation in a larger number of patients on measurements of ventilation and gas exchange in COPD could potentially build on these initial observations. A longer study duration would also be required to adequately assess the impact of increased ventilated lung volume on patient symptoms and enhance understanding of this mechanistic relationship with clinical outcomes and exacerbation frequency. The impact of improved ventilation with IND/GLY on patient-reported outcomes or gas tension was not captured in this study. This mechanistic study in moderate to severe COPD patients (without any predisposition for hyperinflation) confirms our understanding of ventilation and perfusion changes with IND/GLY in a more general COPD patient population. These findings expand our understanding from previously shown data in COPD with significant hyperinflation (residual volume > 135% predicted) at baseline [7]. That study used dynamic gadolinium-enhanced and phase-resolved functional lung MRI [7] and demonstrated IND/GLY to improve microvascular blood flow and regional ventilation. Lung deflation with IND/GLY also improved biventricular cardiac filling and cardiac output, with a concomitant improvement in patient-reported health status and symptoms [21]. Further studies assessing the impact on other parameters such as quality of life, health status, or exercise tolerance are warranted. Although already proposed for asthmatics [19], these studies would help to establish a MCID for changes in ventilated lung volume as assessed by lung MRI for patients with COPD, and could be better powered to specifically examine V/Q matching and relationships with traditional pulmonary function tests. Furthermore, a registration-based approach to estimating V/Q match in future studies could also be explored.

In this study, ventilation increase was measured using the ventilation imaging method of hyperpolarized gas (^3^He) MRI; we used ^3^He, however ^129^Xe can also be used for ventilation MRI. While this technique is highly sensitive, samples the entire lung volume, and does not involve harmful ionizing radiation [1], clinical improvement for patients will ultimately depend on the translation of the results into specific treatment decisions in clinical practice. Additionally, the applied image analysis focused on global and lobar comparisons of the V/Q index; methods that focus on a voxel-wise comparison may further elucidate the mechanistic response and interpretation of these lung MRI data [16]. The combination of ^3^He plus Gd-enhanced MRI modality was successfully embedded in a cross-over study design. The MRI approach demonstrated sufficient sensitivity to elucidate the pharmacodynamic action of IND/GLY. Hyperpolarized gas imaging may help to sensitively detect ventilation abnormalities in different phenotypes of COPD as recently shown for patients with more bronchitis-predominant phenotype [28].

Mummy et al. [28] assessed regional gas exchange ^129^Xe MRI alone in patients with COPD, before and after combination bronchodilator, where ventilation increased after bronchodilator but regional gas exchange did not. The gas exchange ^129^Xe imaging method employed by Mummy et al. measures the ratio of ^129^Xe in the red blood cells to ^129^Xe in the ventilated airspaces. That method inherently can only access the regions of ventilated lung and mechanistically does not show regions of V/Q mismatch. Our study imaged perfusion using an injected contrast agent and a global or lobar ratio of ventilation over perfusion was used to assess V/Q. Hence, in our study we were able to show that a broad and heterogeneous population of COPD patients benefit from dual bronchodilation in terms of improvements in ventilation homogeneity and V/Q mismatch. Apart from these fundamental methodological differences, the differing results between that study [28] and ours may also relate to the definition of the clinical cohort such as excluding patients with gross emphysema in our work. While degree of emphysema was not correlated with improvements in ventilation parameters following IND/GLY, it must be noted that our study does not determine whether patients with gross emphysema would benefit in a similar fashion.

As to our knowledge this is the first time that within such a short treatment duration of 7 days the ^3^He plus Gd-enhanced 1H MRI method has been shown to reliably assess regional/global V/Q matching in COPD patients, and perhaps further response to IND/GLY may have been detected at a later timepoint. Baselines demonstrated a reproducible phenotype of these otherwise heterogenous COPD patients. Low N clinical studies (here N = 28 completers) are extremely valuable and have the needed sensitivity for detection of therapeutic benefit in short treatment time frames. The MRI approach relegated to specialty imaging centers provides an opportunity to inform early drug development of new treatment modalities for obstructive pulmonary diseases providing visualization and quantitation of the regional and global V/Q status.

In conclusion, dual bronchodilation with IND/GLY improved global and regional lung ventilation, and improved global V/Q after 1 week of treatment. Additional findings included improved gas exchange as evidenced by a trend towards increased DL_CO_. These observations may help to explain the benefit shown with IND/GLY in terms of improved patient-reported symptom improvements. A potential opportunity for early intervention with IND/GLY to rebalance the V/Q ratio and improve oxygenation in the appropriate patients may improve symptoms and quality of life for patients with COPD. Conclusively determining whether global %VV measured by hyperpolarized gas MRI could be a sensitive parameter to predict patient benefit (e.g. in terms of reduced risk of exacerbations or improvements in symptom scores and quality of life) requires future research.

## Supplementary Information

**Additional file 1:** Online data supplement and supplementary videos.

## Data Availability

Novartis is committed to sharing with qualified external researchers, access to patient-level data and supporting clinical documents from eligible studies. These requests are reviewed and approved by an independent review panel on the basis of scientific merit. All data provided are anonymized to respect the privacy of patients who have participated in the trial in line with applicable laws and regulations. The trial data availability is according to the criteria and process described at https://www.clinicalstudydatarequest.com/.
