# Supplementary material for: Effect of indacaterol/glycopyrronium on ventilation and perfusion in COPD: a randomized trial
Source: Respir Res. 2022 Feb 10;23:26. doi: 10.1186/s12931-022-01949-3 (PMC8832861; doi:10.1186/s12931-022-01949-3)
Supplement: Supplementary file 1 — Additional file 1: Online data supplement and supplementary videos. [file 12931_2022_1949_MOESM1_ESM.zip › 12931_2022_1949_MOESM1_ESM.docx]

# Online Data Supplement

**Effect of Indacaterol/Glycopyrronium on Ventilation and Perfusion in COPD: A Randomized Trial**

Dave Singh, Jim M. Wild, Dinesh Saralaya, Rod Lawson, Helen Marshall, Jonathan Goldin, Matthew S. Brown, Konstantinos Kostikas, Kristin Belmore, Robert Fogel, Francesco Patalano, Anton Drollmann, Surendra Machineni, Ieuan Jones, Denise Yates, and Hanns-Christian Tillmann

# Methods

## Participants

Inclusion criteria

1. Written informed consent was obtained before any assessment was performed.
2. Male and females with chronic obstructive pulmonary disease (COPD) aged 40 years and above.
3. Smokers and ex-smokers who had a smoking history of at least 10 pack years. Smokers were defined as any patient who reported tobacco use within the last month.
4. Patients with a diagnosis of moderate-to-severe COPD according to GOLD 2015 criteria. Patients with airflow limitation indicated by a post-bronchodilator forced expiratory volume in one second/forced vital capacity (FEV_1_/FVC) ratio <0.70 and by a post-bronchodilator FEV_1_ ≥30–<80%
   - Post-bronchodilator refers to 1 h (± 5 min) after sequential inhalation of 84 μg ipratropium bromide (or equivalent dose) and 400 μg salbutamol/360 μg albuterol (or equivalent dose). Spacer devices were not permitted during reversibility testing.
5. Patients had to weigh ≥45–≤100 kg to participate in the study.
6. Able to communicate well with the investigator, to understand and comply with the requirements of the study.

Exclusion criteria

1. Use of other investigational drugs at the time of enrollment, or within 5 half-lives of enrollment, or within 30 days, whichever is longer; or longer if required by local regulations.
2. History of hypersensitivity to any of the study drugs or to drugs of similar chemical classes.
3. Any significant medical condition that, in the opinion of the investigator, may compromise patient safety, patient compliance, interfere with evaluations, or preclude completion of the trial. For example, COPD exacerbations within the 6 weeks prior to screening requiring oral steroids and/or antibiotics.
4. A history of clinically significant electrocardiogram (ECG) abnormalities (in the opinion of the investigator), or any of the following ECG abnormalities at screening:

- PR >200 msec
- QRS complex >120 msec
- QTcF >450 msec

1. Known family history or known presence of long QT syndrome.
2. Known history of or current clinically significant arrhythmias.
3. Patients with symptomatic heart failure (New York Heart Association [NYHA]line Grade II or greater).
4. Patients requiring oxygen therapy for chronic hypoxemia (excluding acute COPD exacerbation). This was typically patients requiring oxygen therapy >15 h per day delivered by home oxygen cylinder or concentrator or non-invasive positive pressure ventilation.
5. ≥25% emphysematous changes (determined from quantitative image analysis of an inspiratory high-resolution computed tomography [HRCT] scan) in total lung as determined at screening or on a historical HRCT scan not older than 6 months.
6. Patients who had experienced a lower respiratory tract infection within 6 weeks prior to screening, or significant illness, which had not resolved within two weeks prior to initial dosing.
7. Patients with concomitant pulmonary disease, e.g. pulmonary tuberculosis (unless confirmed by chest x-ray to be no longer active) or clinically significant bronchiectasis.
8. Patients with a history of asthma, indicated by (but not limited to):

- Onset of respiratory symptoms suggestive of asthma (such as cough, wheezing, shortness of breath) prior to age 40 years.
- History of a diagnosis of asthma.

1. HbA1c >8% at screening to identify patients with poorly controlled Type I or Type II diabetes.
2. Pregnant or nursing (lactating) women, where pregnancy is defined as the state of a female after conception and until the termination of gestation, confirmed by a positive serum hCG laboratory test at screening or urine hCG test prior to initial dosage.
3. Women of child-bearing potential, defined as all women physiologically capable of becoming pregnant.
4. Patients receiving long-acting muscarinic antagonists (LAMA), short-acting muscarinic antagonists (SAMA), long-acting β_2_-agonists (LABA), short-acting β_2_-agonists (SABA), LABA/inhaled corticosteroid (ICS) fixed combinations, SABA/SAMA fixed combinations, oral phosphodiesterase-IV inhibitors, or xanthines.
5. Patients receiving any COPD-related medications should be excluded if, in the opinion of the investigator, the patient could not safely be withdrawn from these medications at Visit 2 and to follow the adjustment to the treatment program.
6. Patients receiving selective serotonin re-uptake inhibitors, intra-nasal corticosteroids, ICS, H_1_-antagonists, or inactivated influenza, pneumococcal, or any other inactivated vaccine should be excluded unless the medication has been stable for the specified period and the stated conditions have been met.
7. Patients unable to successfully use a dry powder inhaler device or perform spirometry, multiple breath nitrogen washout/lung volumes, and diffusing capacity for carbon monoxide (DL_CO_) measurements.
8. History of drug or alcohol abuse within the 12 months prior to drug administration, or evidence of such abuse as indicated by the laboratory assays conducted at screening.
9. Impaired renal function during screening defined as an estimated glomerular filtration rate (eGFR) at screening <30 mL/min/1.73 m^2^, calculated using the simplified Modification of Diet in Renal Disease (sMDRD) equation due to potential issue with administration of Gd-DTPA used as the magnetic resonance imaging (MRI) contrast agent.
10. For a patient to be enrolled in the study the following exclusion criteria relating to MRI will apply; specifically patients with contraindications to MRI, including those listed below are excluded from this study:

- Brain aneurysm clip
- Implanted neural stimulator
- Implanted cardiac pacemaker or defibrillator, or presence of intracardiac wires
- Prosthetic heart valves
- Cochlear implant
- Renal transplant
- Liver transplant
- Ocular foreign bodies that might be ferromagnetic (e.g. metal shavings)
- Other implanted medical devices (e.g. insulin pumps)
- Metal shrapnel or bullets still in the body
- Severe claustrophobia
- Body mass index greater than 40 kg/m^2^, as well as body habitus to ensure positioning and patient comfort within the constraints of the MRI scanner for the duration of the imaging protocol. Investigators should use their best judgment to assure that patients’ size will not interfere with MRI imaging at any of the specified time-points.
- Tattoos (as determined by the investigator and imager)

1. History of malignancy of any organ system (other than localized basal cell carcinoma of the skin), treated or untreated, within the previous 5 years, regardless of whether there is evidence of local recurrence or metastases.
2. A positive hepatitis B surface antigen, HIV, or hepatitis C test result.

## Study design

This was a double-blind, randomized, placebo-controlled, two-period, crossover study in patients with moderate-to-severe COPD. The study consisted of a 2-week screening period, a 7–9 day run-in, a baseline visit, a double-blind (up to 10 days) treatment period I followed by a washout period, before crossing over to a double-blind (up to 10 days) treatment period II, and an end-of-study visit. At the run-in visit, prohibited COPD medications were removed. These included LAMA, SAMA, LABA, SABA, LABA/ICS fixed combinations, SABA/SAMA fixed combinations, oral phosphodiesterase-IV inhibitors, and xanthines. Patients who entered the study on ICS were permitted to remain on their background ICS. As-needed SABA rescue medication was available to patients throughout the duration of the study, but was withheld for 6 hours prior to study assessments.

Patient tolerance for long-acting bronchodilator removal was assessed during the run-in period by the study investigator prior to baseline assessments. To minimize patient burden, MRI and lung function assessments were not performed on the same day. Patients had three MRI sessions, at baseline and on Day 7 of treatment periods I and II – approximately 10 min for ^3^He MRI and 10 min for proton MRI for a total of approximately 30 min per session, including the set-up and completion of 6 sequences. Actual MRI assessments commenced 2 hours after patient administration at the site. All other assessments, in particular spirometry and DL_CO_, were done the day after the MRI assessments, i.e. Day 8 of treatment during periods I and II.

## Treatment administration regime

The current recommended dose of this dual bronchodilator is 110 μg indacaterol maleate/50 μg glycopyrronium bromide (IND/GLY) once per day. Study treatment capsules (IND/GLY or matched placebo) were delivered via the Concept1 inhaler device. In each treatment period, inhalation of the first dose of study drug by the patient was performed under the supervision of study personnel to ensure correct inhalation. On study visit days, the drug was taken in the presence and under the guidance of study site personnel. On all other days, the study drug was taken at home. The patients were advised to contact the investigator site by telephone in case of questions during home use. On study visit days that included an MRI assessment, the dose was administered at the imaging clinic.

## Randomization and treatment assignment

Randomization numbers were assigned in ascending, scrambled order to eligible patients. The investigator entered the randomization number on the Case Report Form (CRF). The randomization numbers were generated using the following procedure to ensure that treatment assignment was unbiased and concealed from patients and investigator staff. The production of a treatment allocation card was the responsibility of Novartis drug supply management using a validated system that automated the random assignment of treatment arms to randomization numbers in the specified ratio. The randomization scheme for patients were reviewed and approved by a member of the Novartis IIS Randomization Group. Patients had to meet the inclusion/exclusion criteria to be randomized. If a randomization number was allocated to a patient who subsequently did not meet the study eligibility criteria, then the patient was a screen failure. The allocated randomization number was reused in the study.

## Treatment blinding

This was a double-blind study: patients, investigator staff, persons performing the assessments, and data analysts remained blinded to the identity of study treatments. The identity of the treatments was concealed by the use of study drugs that were all identical in packaging, labeling, schedule of administration, appearance, and odor. Randomization data were kept strictly confidential, and were accessible only to authorized personnel, until unblinding of the trial at the end of the study.

## Study assessments

***HRCT acquisition and analysis***

Non-contrast high-resolution CT images were acquired with the subject in a supine position with breath-holding at total lung capacity (TLC). Multicenter CT scanning was calibrated with a phantom (COPD Gene design) for each patient screening. All CT scans were transferred electronically to the imaging CRO MedQIA (Los Angeles, USA) and assessed for eligibility based on a quantitative emphysema score (percentage of voxels below a CT attenuation threshold), which had to be less than 25% of the whole lung and also qualitatively evaluated to ensure the absence of significant bronchiectasis

***MRI acquisition***

Hyperpolarized ^3^He gas ventilation and ^1^H gadolinium contrast-enhanced lung perfusion imaging were performed at baseline and on Day 7 of each treatment period (2 h post-dose).

MRI was performed on a 1.5T system (GE HDx, GE Healthcare, Milwaukee, WI) equipped for hyperpolarized gas MRI. For ventilation imaging (1), patients were positioned supine in a ^3^He transmit-receive coil (Clinical MR Solutions, Brookfield, WI) and a mix of 200 mL hyperpolarized ^3^He gas (polarization ~25%, GE Healthcare, Amersham, UK) and 800 mL N_2_ was inhaled from functional residual capacity (FRC). ^3^He ventilation images were acquired at breath-hold with the following parameters: 3D balanced steady state free precession (bSSFP) sequence, coronal, matrix=100x80, field of view (FOV)=40–48 cm, 5 mm slices, full lung coverage (approx. 48 slices), bandwidth (BW)=167 kHz, echo time/repetition time (TE/TR)=0.6/1.9 ms, flip angle (FA)=10⁰. Within the same breath-hold, ^1^H anatomical images of the same imaging volume were acquired with the following parameters: 3D spoiled gradient echo (SPGR) sequence, coronal, matrix=100x100, 5 mm slices, BW=167 kHz, TE/TR=0.6/1.4 ms, FA=5⁰. The scan time was approximately 14 sec. For perfusion imaging, patients were repositioned into a ^1^H 8 element chest coil array (GE, Milwaukee, WI). In order to calculate pre-contrast T_1_ maps, three 3D ^1^H image volumes with different flip angles were acquired during separate inspiratory breath-holds, with the parameters: 3D SPGR, coronal, matrix=200x80, FOV=48 cm, 4 mm slices, full lung coverage (approx. 64 slices), BW=125 kHz, TE/TR=0.9/2.85 ms, FA=2º, 10º and 30º. ^1^H MRI images were acquired during inspiratory breath-hold with a 3D SPGR sequence using view sharing (TRICKS) (2) and parallel imaging (SENSE R=2) (3) with sequence parameters: coronal, matrix=200x80, FOV=48 cm, 10 mm slices, full lung coverage (approx. 24 slices), BW=250 kHz, TE/TR=1.1/2.5 ms, FA=30⁰, temporal phases=36, and temporal resolution of 0.5 s per 3D volume. The contrast agent was delivered as a 0.05 mL/kg bolus of Gadovist at a flow rate of 4 mL/sec, followed by a 20 mL saline flush at the same flow rate.

**Ventilation image analysis**

The whole lung volumes for right and left were segmented by applying intensity thresholding on the thoracic ^1^H lung MR images. To achieve lobar segmentation, the lungs and lobes were first segmented semi-automatically on the patient’s CT scan, where the inter-lobar fissures could be more clearly identified. The lobar segmentations from the CT scan were then transformed to the right and left lungs through image registration of the CT and proton MRI scans. Segmented lung and lobe masks were mapped to the ^3^He MRI, no registration was applied in this step since the images were acquired during the same breath-hold (4). Within the segmented lung fields (excluding the central airways based on CT scan), ^3^He MRI intensity values were normalized (up to 99.5^th^ percentile) within each scan by applying the N4 Bias Field Correction algorithm and remapping such that 0.0 value represented “no ventilation” and 1.0 value represented “maximum ventilation”.

Across all MRI time points, the same CT scan was used for registration and a consistent intensity thresholding was performed on the normalized ^3^He images within the segmented lung regions, and voxels above the threshold (0.3) were labeled as ventilated. The percentage ventilated lung volume (%VV) was computed as the number of ventilated voxels relative to the total number of voxels in the segmented lung (5). The ventilation threshold was initially set empirically based on an image review by a radiologist with experience reading ^3^He ventilation images. The normalization approach, based on the intensity maximum within the lungs, is based upon the assumption that if the maximum changes between timepoints of scanning subject, then the entire histogram scales approximately linearly.

**Perfusion image analysis**

*Pulmonary perfusion* was derived using dynamic contrast-enhanced (DCE) lung MRI imaging (6). Voxel-wise contrast concentration time curves (CTC) were obtained from DCE and variable flip angle MRI scans (7), registration was not performed among scans. Mean CTC at the pulmonary artery was obtained as the arterial input function. A constant of 0.4 was used (tissue density of 0.4 g/mL) when converting the flow unit from mL/min to mL/100 g/min^3^.

Pulmonary blood volume was then derived from the arterial input function and the CTCs. Similar to the computation of %VV, percentage perfused lung volume (%PV) was calculated by first normalizing (up to 99.5^th^ percentile) the perfusion image to 1.0 by dividing by the maximum value of the perfusion dataset, i.e. the measure is converted from mL/100 g_(lung tissue)_/min to become unitless. Voxels were then defined as perfused if their normalized value was above an empirically determined threshold (0.05). %PV was computed as the number of perfused voxels relative to the total voxel count within the segmented lung, i.e. the fraction of voxels that were perfused within the lung. Major vessels were not excluded. The right and left lungs were segmented by first manually segmenting the thoracic ^1^H images. Affine registration was then performed between the proton scan and the first DCE scan, and the segmented lung was registered from the proton scan to the DCE scan. The perfusion threshold was set empirically based on review of scans from normal and abnormal patients by a radiologist.

The *MRI based ventilation volume to perfusion volume ratio* denoted as V/Q was calculated using the global and regional ratios of scaled ventilation and perfusion images. For this study, the ratio was defined as the ratio of ventilation fraction to perfusion fraction. Both fractions are unit-free percentages. The ratio yielded a value of 1.0 if the proportions of lung perfused and ventilated were the same, with values >1 if a greater proportion of the lung was ventilated, and <1 if a greater proportion of the lung was perfused. This approach did not involve fusion of ventilation and perfusion maps on a per pixel basis, but was instead based on relative proportions of lung involvement derived from the global and lobar analysis of ventilation and perfusion, independently. Future analyses beyond the global ratio may provide more detailed mechanistic insight on the regional ventilation-perfusion response, in particular fusion of the maps would allow more direct assessment of regions where ventilation and perfusion are mismatched.

*Spirometry* measurements followed the ATS/ERS guidelines (8). Briefly, a minimum of 3 acceptable FVC maneuvers were performed. The two largest FVC and FEV_1_ values from 3 acceptable maneuvers did not vary by more than 0.150 L and the highest FEV_1_ and FVC from any of the acceptable curves was recorded. Post-bronchodilator FEV_1_ was recorded 1 h after sequential inhalation of 4 x 21 μg (ex-valve) ipratropium bromide (or equivalent dose). Note: a single dose of ipratropium bromide monohydrate of 21 μg is equivalent to ipratropium bromide water-free 20 μg. Four doses were needed to equate to 84 μg (80 water free μg) and 4 x 100 μg puffs of salbutamol (equivalent to 4 x 90 μg albuterol delivered at the mouthpiece).

*DL_CO_* was performed on Day 8 of each treatment period in line with ATS/ERS guidelines (9). Briefly, once the mouthpiece of the gas analyzer and nose clip were in situ, the DL_CO_ measurement began with an unforced exhalation (6 sec maximum), followed by rapid inhalation by the patient (<4 sec) once the mouthpiece had been connected to the test gases. Breath-hold time was no longer than 4 sec before patients were asked to expire smoothly and fully. An expiration wash-out of approximately 0.75–1.00 L was discarded before using the sample gas volume to measure alveolar carbon monoxide (CO) and tracer gas concentrations, according to the following equation: DL_CO_ = total CO uptake overtime/P_ACO_ = Δ[CO] x V_A_/Δt/P_ACO_.

## Statistical analysis

Sample size was calculated such that using a within-patient standard deviation of 7%, a sample size of 28 completers (14 patients per treatment) would provide ≥80% power to detect a 5% improvement in % global ventilation volume using a 2-sided test at the 10% level of significance. Sample size calculations were performed using nQuery 7.0 software. The sample size of 28 patients also provided ≥80% power to detect a difference of 0.170 L in trough FEV_1_ using a 2-sided test at the 10% level of significance.

All primary and secondary analyses were performed in the pharmacodynamics (PD) population, i.e. all patients with evaluable PD parameter data and no major protocol deviations were included. The primary outcome, percent of global ventilation volume on Day 7, was analyzed using a mixed effects model, including crossover sequence, period, and treatment as fixed effects, and patient factor as a random effect. An unstructured covariance matrix was applied. Final model estimates include a least squares (LS) mean for each treatment, the adjusted mean difference between IND/GLY and placebo, the corresponding 90% two-sided confidence intervals and P value, using placebo as the reference treatment. The model described for the primary outcome was also applied to all secondary and exploratory outcomes. For spirometry parameters, the model included time (15 min, 1 h, and 2 h post-dose) as a fixed effect. No adjustments for multiplicity were applied for this exploratory study and missing data were not imputed for the primary analysis.

Summary statistics are provided for all background and demographic variables by treatment sequence. Correlation analysis using Spearman’s rank correlation analyses was also employed.

## Regulatory and ethical approval

The study was conducted in accordance with the ICH Harmonized Tripartite Guidelines for Good Clinical Practice, with applicable local regulations (including European Directive 2001/20/EC, US Code of Federal Regulations Title 21, and Japanese Ministry of Health, Labor, and Welfare), and with the ethical principles of the Declaration of Helsinki. Ethical approval was obtained from the Institutional Review Board/Independent Ethics Committee of the participating study sites.

# Tables

# Table E1. Overall incidence of adverse and serious adverse events (safety set, n=31)

| **Parameter** | **IND/GLY (n=31)** | **Placebo (n=31)** |
| --- | --- | --- |
| **Number of patients with at least one AE, n (%)** | 5 (16.1) | 4 (12.9) |
| Chronic obstructive pulmonary disease | 2 (6.5) | 0 (0.0) |
| Productive cough | 1 (3.2) | 1 (3.2) |
| Atrial fibrillation | 0 (0.0) | 1 (3.2) |
| Cough | 1 (3.2) | 0 (0.0) |
| Dizziness | 0 (0.0) | 1 (3.2) |
| Dyspnea | 0 (0.0) | 1 (3.2) |
| Facial pain | 1 (3.2) | 0 (0.0) |
| Femoral neck fracture | 0 (0.0) | 1 (3.2) |
| Headache | 1 (3.2) | 0 (0.0) |
| Migraine | 1 (3.2) | 0 (0.0) |
| Pain in extremity | 1 (3.2) | 0 (0.0) |
| Vomiting | 1 (3.2) | 0 (0.0) |
| **Number of patients with at least one SAE, n (%)** | 0 (0.0) | 2 (6.5) |
| Atrial fibrillation | 0 (0.0) | 1 (3.2) |
| Femoral neck fracture | 0 (0.0) | 1 (3.2) |

Values given are n (%).

AE = adverse event; SAE = serious adverse event

# Figure S1. Distribution of ^3^He within the lung by MRI: quantitative percentage ventilated lung volume assessment for Patient 2

Shown are example ^3^He MRI image segmentations of the lungs acquired during the placebo and treatment periods from a patient demonstrating the effect of IND/GLY compared with placebo. *Upper panels:* Source images showing the distribution of hyperpolarized ^3^He gas within the lung. 2D coronal slices from back to front (upper left to lower right) were acquired for volumetric assessment after 7 days of placebo and IND/GLY treatment in the respective treatment periods. *Centre panels:* Image segmentations with ventilated lung shown in blue and unventilated lung shown in brown (2D slices). *Lower panels:* 3D volume rendering of the image segmentations.


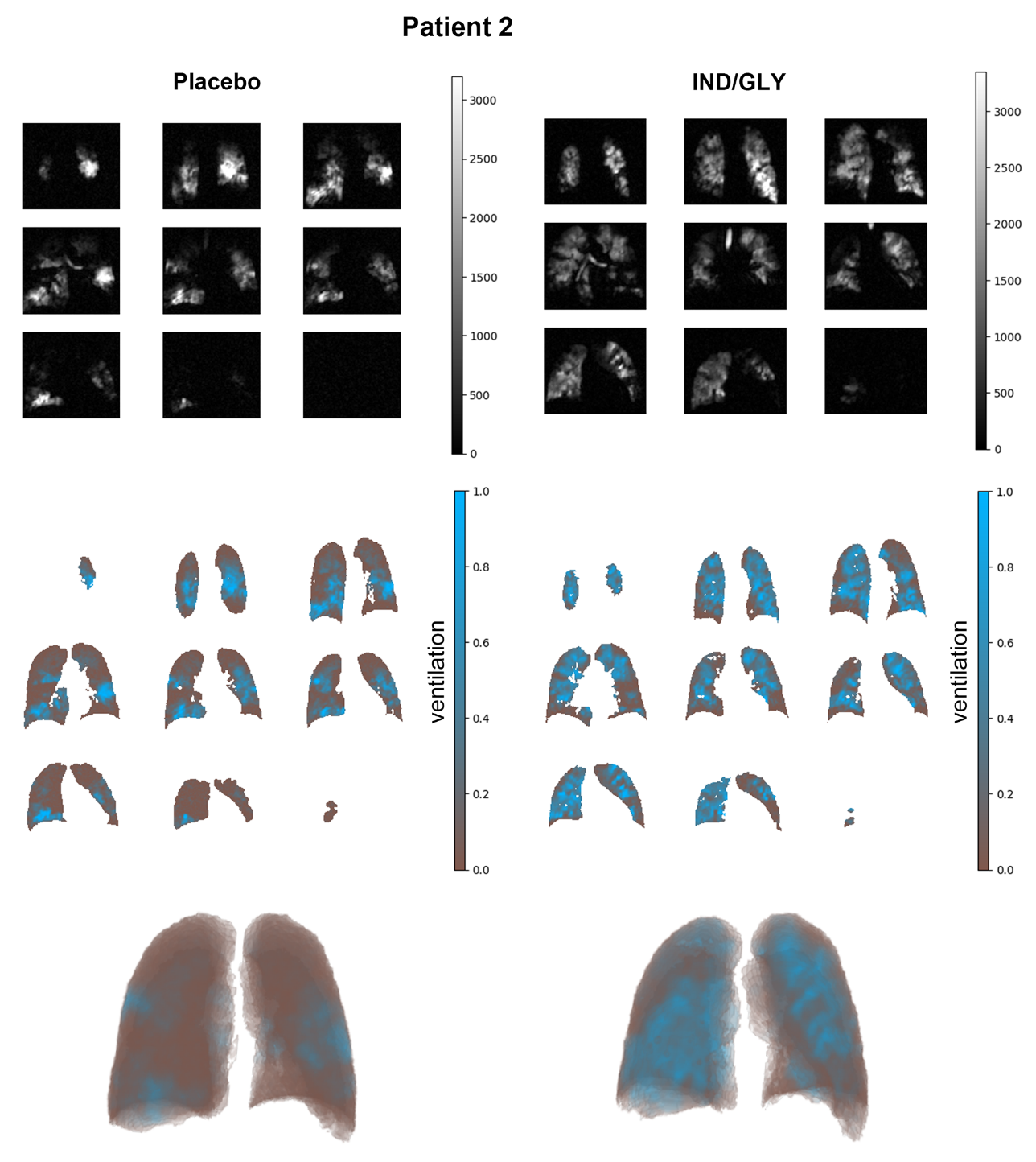


**Supplementary Video 1**: 3D-rendered videos of the entire ventilated lung volume to illustrate improvement in global %VV with IND/GLY versus placebo (Patient 1)
Ventilated lung shown in blue and unventilated lung shown in brown; %VV, percentage ventilated lung volume;
IND/GLY, indacaterol/glycopyrronium

[
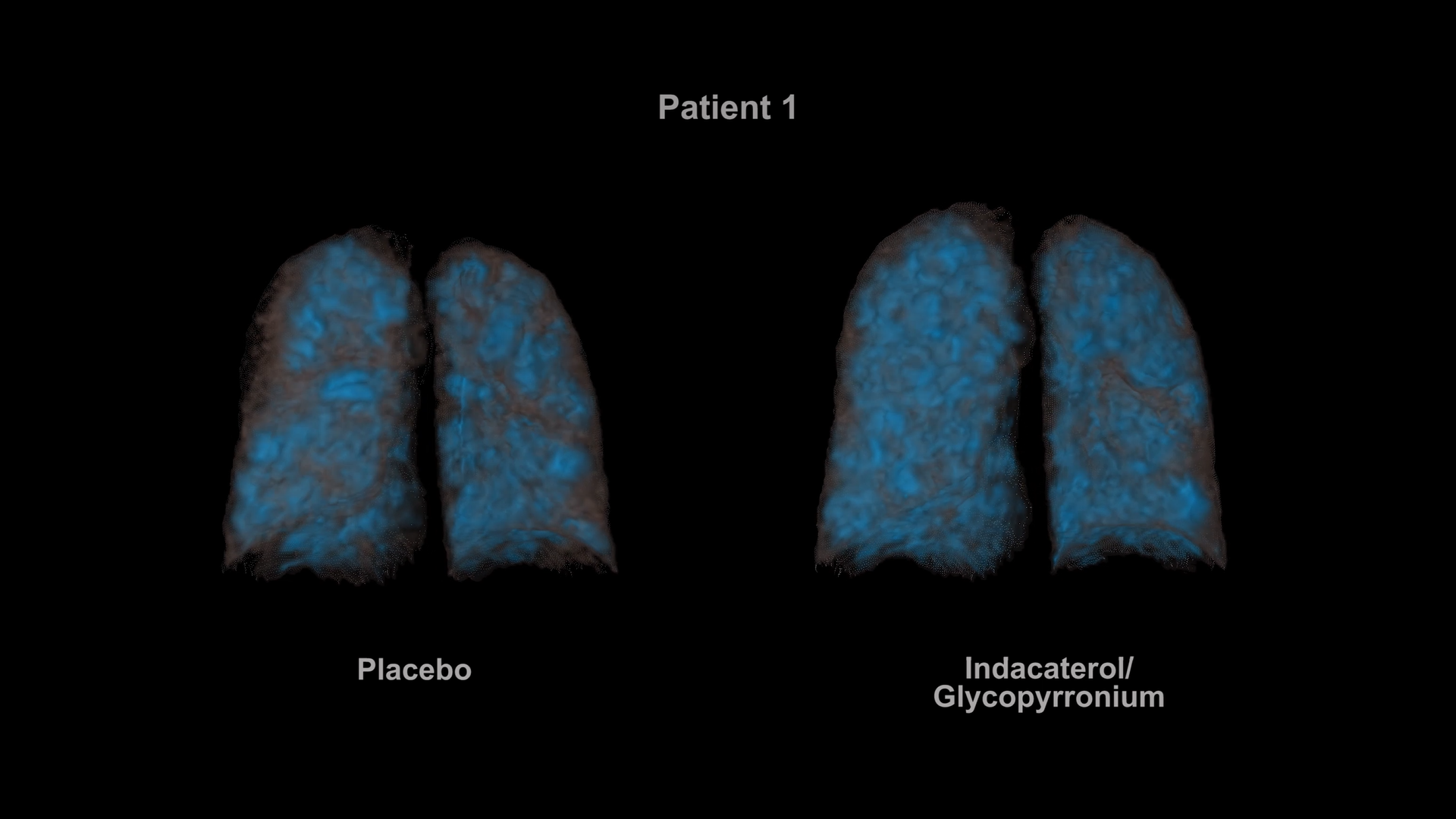
](https://my.novartis.net/:f:/g/personal/katdaaa1_novartis_net/Er7gR_96WVdBoNcayLfpwxoBMs9BD5OcqoLKXndGB4_E0Q?e=Z5FbJQ)

**Supplementary Video 2**: 3D-rendered videos of the entire ventilated lung volume to illustrate improvement in global %VV with IND/GLY versus placebo (Patient 2)
Ventilated lung shown in blue and unventilated lung shown in brown; %VV, percentage ventilated lung volume;
IND/GLY, indacaterol/glycopyrronium

[
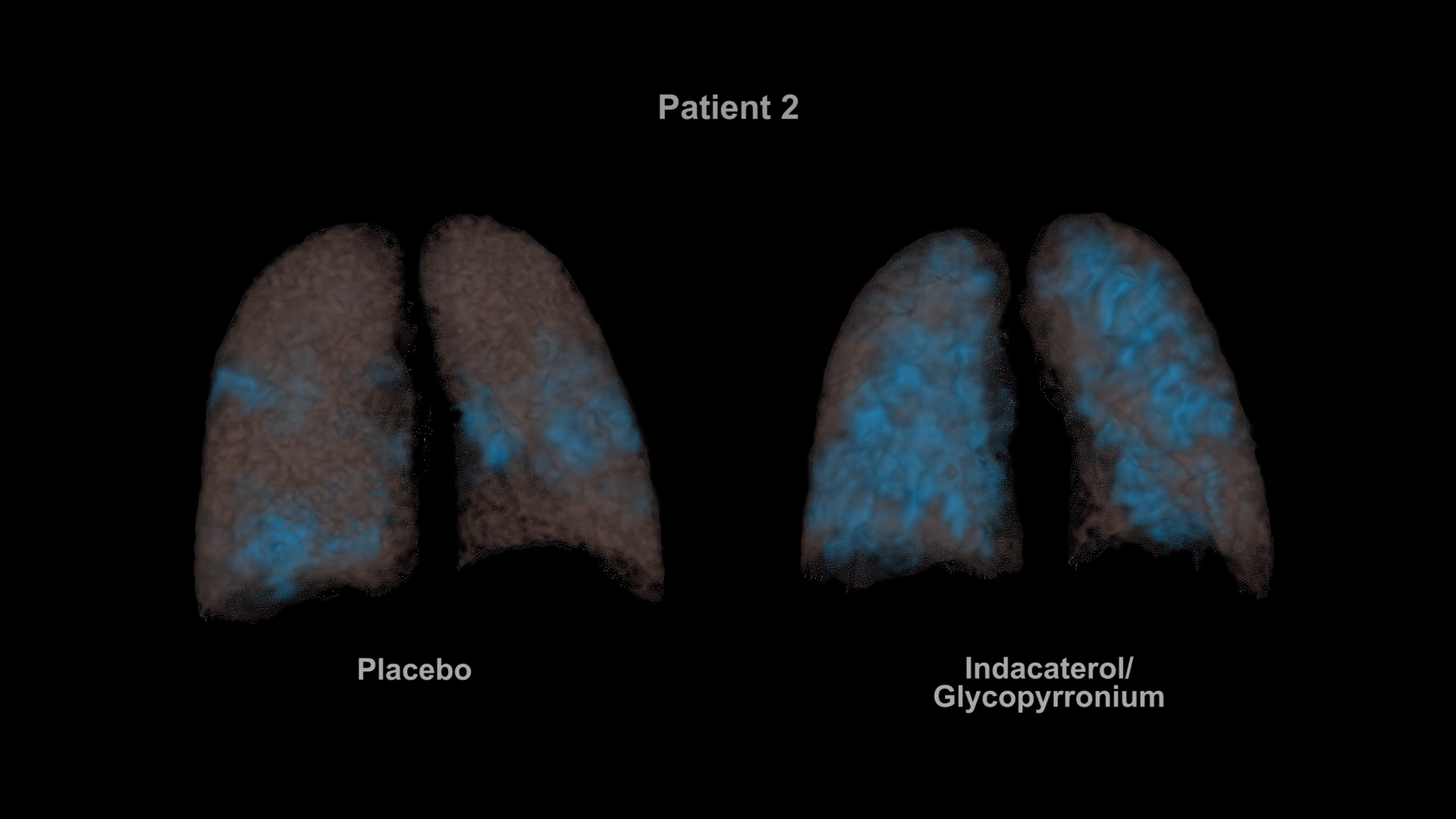
](https://my.novartis.net/:f:/g/personal/katdaaa1_novartis_net/Er7gR_96WVdBoNcayLfpwxoBMs9BD5OcqoLKXndGB4_E0Q?e=uqgZQo)

**Supplementary References**

1. Horn F, Tahir B, Stewart N, Collier G, Norquay G, Leung G, et al. Lung ventilation volumetry with same‐breath acquisition of hyperpolarized gas and proton MRI. NMR in Biomedicine. 2014;27(12):1461-7.

2. Korosec FR, Frayne R, Grist TM, Mistretta CA. Time‐resolved contrast‐enhanced 3D MR angiography. Magnetic Resonance in Medicine. 1996;36(3):345-51.

3. Pruessmann KP, Weiger M, Scheidegger MB, Boesiger P. SENSE: sensitivity encoding for fast MRI. Magnetic resonance in medicine. 1999;42(5):952-62.

4. Wild JM, Ajraoui S, Deppe MH, Parnell SR, Marshall H, Parra‐Robles J, et al. Synchronous acquisition of hyperpolarised 3He and 1H MR images of the lungs–maximising mutual anatomical and functional information. NMR in Biomedicine. 2011;24(2):130-4.

5. Woodhouse N, Wild JM, Paley MN, Fichele S, Said Z, Swift AJ, et al. Combined helium‐3/proton magnetic resonance imaging measurement of ventilated lung volumes in smokers compared to never‐smokers. Journal of Magnetic Resonance Imaging: An Official Journal of the International Society for Magnetic Resonance in Medicine. 2005;21(4):365-9.

6. Østergaard L. Principles of cerebral perfusion imaging by bolus tracking. Journal of Magnetic Resonance Imaging: An Official Journal of the International Society for Magnetic Resonance in Medicine. 2005;22(6):710-7.

7. Zhu X, Li K, Kamaly‐Asl I, Checkley D, Tessier J, Waterton J, et al. Quantification of endothelial permeability, leakage space, and blood volume in brain tumors using combined T1 and T2* contrast‐enhanced dynamic MR imaging. Journal of Magnetic Resonance Imaging: An Official Journal of the International Society for Magnetic Resonance in Medicine. 2000;11(6):575-85.

8. Miller MR, Hankinson J, Brusasco V, Burgos F, Casaburi R, Coates A, et al. Standardisation of spirometry. European respiratory journal. 2005;26(2):319-38.

9. Macintyre N, Crapo R, Viegi G, Johnson D, Van Der Grinten C, Brusasco V, et al. Standardisation of the single-breath determination of carbon monoxide uptake in the lung. European Respiratory Journal. 2005;26(4):720-35.
